# Conjugated Linolenic Acids Induce Ferroptosis in Human and Zebrafish Melanoma Cells

**DOI:** 10.3390/antiox15030360

**Published:** 2026-03-12

**Authors:** Zhuo Zhang, Alice Valembois, Caroline Rosier, Renaud Bonnevie, Ineke Neefs, Aurélien Warnant, Perrine Vermonden, Melissa M. Page, Olivier Feron, Cathy Debier, Yvan Larondelle

**Affiliations:** 1 Louvain Institute of Biomolecular Science and Technology, UCLouvain, 1348 Louvain-la-Neuve, Belgium; zhuo.zhang@uclouvain.be (Z.Z.); alice.valembois@uclouvain.be (A.V.); caroline.rosier@uclouvain.be (C.R.); renaudbonnevie@hotmail.com (R.B.); ineke.neefs@uclouvain.be (I.N.); aurelien.warnant@uclouvain.be (A.W.); melissa.page@uclouvain.be (M.M.P.); yvan.larondelle@uclouvain.be (Y.L.); 2 Wallonia Institute of Food Science and Technology, 6041 Charleroi, Belgium; 3 Institute of Metabolism and Cell Death, Helmholtz Munich, 85764 Neuherberg, Germany; perrine.vermonden@helmholtz-munich.de; 4 Pole of Pharmacology and Therapeutics (FATH), Institut de Recherche Experimentale et Clinique, UCLouvain, 1200 Woluwe Saint-Lambert, Belgium; olivier.feron@uclouvain.be

**Keywords:** conjugated linolenic acids, human melanoma, zebrafish melanoma, ferroptosis, lipid peroxidation

## Abstract

Conjugated linolenic acids (CLnAs) are emerging as promising agents to trigger ferroptosis, a cell death driven by excessive lipid peroxidation, in cancer cells. Given the aggressive nature and treatment resistance of malignant melanoma, exploring CLnAs as therapeutic agents may offer a novel strategy to overcome these challenges. Here, we investigated the toxicity of four CLnA isomers on human (A375, WM266.4) and zebrafish (ZMEL1) melanoma cell lines. We observed a dose-dependent reduction in cell viability across all three tested cell lines. While human melanoma cells were more sensitive to CLnAs than ZMEL1 cells, treatment with ferroptosis inhibitors mitigated cell death in all models, confirming ferroptosis as the consistent primary mechanism of cell death. In addition, chemical inhibitors of ACSL4 and GPX4 modulated CLnA toxicity, further substantiating the ferroptotic mechanism by highlighting the role of these key regulators. Furthermore, fatty acid analysis revealed that CLnAs were effectively incorporated into phospholipids, generating substrates for lethal lipid peroxidation. At the transcriptional level, CLnA treatment significantly upregulated the pro-ferroptotic gene *acsl4a* in ZMEL1 cells. Overall, our study identifies specific CLnAs as potent ferroptosis inducers in both human and zebrafish melanoma cells and underscores the translational relevance of the zebrafish model based on a shared ferroptotic mechanism.

## 1. Introduction

Skin cancer is among the most common cancers worldwide, with keratinocyte skin cancers, basal cell carcinoma, squamous cell carcinoma, and malignant melanoma being the primary types [1]. Among these, malignant melanoma is highly aggressive and deadly [2]. Melanomas exhibit high mutation rates, the BRAF oncogene being frequently mutated and contributing to rapid tumour growth and treatment resistance [2,3]. The newly diagnosed cases of melanoma have been rising steadily worldwide, with projections suggesting an increase of more than 50% between 2020 and 2040 [4]. Although treatments such as the BRAF inhibitor Vemurafenib and immune checkpoint blockers bring hope to melanoma patients, primary lack of response and acquired treatment resistance remain urgent challenges [5,6,7].

Conjugated linolenic acids (CLnAs) are a group of isomers of α-linolenic acid (ALA) characterized by at least two conjugated double bonds (Figure S1). These fatty acids are predominantly found in the seeds of a limited number of plants [8]. For example, punicic acid (PunA, C18:3 c9, t11, c13), α-eleostearic acid (α-ESA, C18:3 c9, t11, t13) and jacaric acid (JA, C18:3 c8, t10, c12) account, respectively, for up to 80% of pomegranate seed oil, approximately 50% of *Ricinodendron heudelotii* seed oil [9] and approximately 35% of the blue jacaranda seed oil [10]. CLnAs have shown strong anti-carcinogenic potential in multiple experimental models [11]. An early study reported that tung oil, which has a high content of α-ESA (approximately 80% of total fatty acids), exerts intense cytotoxic effects on DLD-1 colorectal adenocarcinoma cells, HepG2 hepatoma cells and A549 lung carcinoma cells [12]. Subsequent research confirmed the strong cytotoxicity of α-ESA in several colon cancer cell lines [13]. Similarly, JA significantly inhibits leukaemia cell proliferation [14] and was recently shown to suppress breast cancer cell proliferation [15]. PunA also exhibits cytotoxicity towards HCT-116 colorectal and FaDu hypopharyngeal cancer cell lines [16], as well as various prostate cancer cell lines [17]. Interestingly, PunA, α-ESA, and JA are highly toxic to proliferative Caco-2 colorectal cancer cells, while the toxicity is significantly decreased when Caco-2 cells have differentiated into epithelial cells forming a functional intestinal barrier [18]. Furthermore, α-ESA does not impair the growth of normal human liver cells, while effectively inhibiting the development of breast cancer cells [19]. Collectively, these findings suggest that CLnA toxicity is preferentially directed toward cancer cells without affecting normal cells. Despite extensive evidence of CLnA cytotoxicity in various cancers, its effects on melanoma cells remain largely unexplored.

CLnAs were initially claimed to induce apoptosis in cancer cells [19,20]. However, a growing body of research has indicated that CLnAs induce cancer cell death through ferroptosis. Ferroptosis is an iron-dependent form of non-apoptotic regulated cell death caused by the unrestrained accumulation of lipid hydroperoxides in cell membranes, leading to lethal membrane damage [21]. An early study already revealed that α-ESA is cytotoxic to human monocytic leukaemia cells through a mechanism involving lipid peroxidation [22]. More recently, α-ESA was shown to induce ferroptosis in triple-negative breast cancer cells [23], β-ESA in fibrosarcoma HT-1080, brain neuroblastoma SK-N-SH, and clear-cell renal carcinoma 786-O cells [24], PunA in hypopharyngeal, colorectal and prostate cancer cells [16,17], and JA in both triple-negative and luminal A breast cancer cell lines [15]. Yet, whether this ferroptotic mechanism extends to melanoma cells remains to be elucidated.

Cancer cells exhibit an elevated need for fatty acids, which are essential for energy production and the synthesis of new cell membranes to sustain cell proliferation [25,26]. The introduction of high amounts of peroxidable polyunsaturated fatty acids (PUFAs) is expected to enhance lipid hydroperoxide production in cancer cells, thereby predisposing them to ferroptosis [27]. Acyl-CoA synthetase long-chain family member 4 (ACSL4) catalyses the activation of PUFAs into PUFA-CoAs, and functions as a ferroptosis inducer [28,29]. PUFA-CoAs can be incorporated into phospholipids (PLs) within lipid membrane structures, through the activity of multiple lysophosphatidylcholine acyltransferases (LPCATs) [30]. In contrast, antioxidant enzymes that function at the lipid membrane act as ferroptosis inhibitors. This is particularly the case for glutathione peroxidase 4 (GPX4), a monomeric selenoenzyme that reduces toxic phospholipid hydroperoxides (PLOOH) to non-toxic phospholipid alcohols (PLOH), using the reduced form of glutathione as a co-substrate. Through its action, GPX4 prevents the accumulation of hydroperoxides in the membrane [27,31]. ACSL4 and GPX4 have thus emerged as valuable targets in cancer therapy [32,33]. Accordingly, these enzymes were selected for mechanistic studies aiming at evaluating the involvement of the ferroptosis process in the cytotoxic effects of CLnAs [34].

This study employed three melanoma cell lines, two of human origin (A375, WM266.4) and one of zebrafish origin (ZMEL1). Including both human and zebrafish melanoma cells helped to overcome the limitations of a single-species model and enhanced the translational relevance of our findings. The A375 melanoma cell line, derived from a primary tumour, known for its high angiogenic and metastatic potential, exhibits rapid tumour growth and invasive behaviour [35], making it a well-established model for studying melanoma progression and therapeutic responses. Similarly, the WM266.4 cell line, originating from a metastatic lymph node, is highly metastatic and frequently used to investigate aggressive melanoma behaviour and the effectiveness of anti-cancer treatments. In parallel, the zebrafish melanoma cell line ZMEL1 offers a distinct advantage: its gene expression profile closely mirrors that of human melanoma cell lines while providing an opportunity to explore ferroptosis in a non-mammalian context [2,36,37]. Beyond this, the use of ZMEL1 is an essential step toward using zebrafish as an in vivo model for melanoma research.

We first evaluated the toxicity of CLnAs on these melanoma cell lines. To elucidate the underlying cell death mechanism, we examined the impact of combining CLnAs with ferroptosis, necroptosis or apoptosis inhibitors. In addition, we used chemical inhibitors of ACSL4 and GPX4 to evaluate their roles in the CLnA-induced cell death process. Given the lower sensitivity of ZMEL1 cells to CLnAs, this zebrafish-derived line was further used to investigate CLnA incorporation and metabolic processing in melanoma cells. ZMEL1 cells were also used to assess the expression levels of the *acsl4* and *gpx4* genes in the presence of CLnAs. To our knowledge, this is the first study exploring the anti-melanoma potential of CLnAs, providing a new direction for melanoma treatment.

## 2. Materials and Methods

### 2.1. Cell Culture

The human melanoma cell lines A375 and WM266.4 were kindly gifted by Professor Bénédicte Jordan (LDRI, UCLouvain, Belgium). The zebrafish melanoma cell line ZMEL1 was kindly provided by the Memorial Sloan Kettering Cancer Center (New York, NY, USA). Human melanoma cells were cultured at 37 °C with 5% CO_2_, in RPMI-1640 medium (21875034, Gibco, Grand Island, NY, USA), supplemented with 10% foetal bovine serum (F7524-500, Merck, Darmstadt, Germany) and 5% penicillin-streptomycin (15140122, Gibco). ZMEL1 cells were maintained at 28.5 °C with 5% CO_2_, in Dulbecco’s Modified Eagle’s Medium (DMEM) (L0102-500, VWR, Radnor, PA, USA), supplemented with 10% foetal bovine serum, 5% penicillin-streptomycin and 5% Glutamax (35050-038, Gibco). Cells were regularly monitored for mycoplasma contamination.

### 2.2. Cell Viability Test

***Impact of fatty acids on melanoma cell viability.*** Before being tested on cells, all fatty acids were conjugated to bovine serum albumin (BSA, A7030-100G, Sigma, Tokyo, Japan) in phosphate-buffered saline (PBS, P4417-100TAB, Sigma). Briefly, BSA was pre-incubated in PBS at 37 °C for 1 h and subsequently mixed with the fatty acids to yield a final fatty acid stock concentration of 5 mM with a FA:BSA molar ratio of 4:1. The mixtures were flushed with argon and incubated at 37 °C with periodic vortexing for up to one week to ensure complete dissolution. Once fully dissolved, the solutions were sterile filtered (0.22 µm) to serve as the stock solutions. The resulting stock solutions were then diluted in culture medium to different working concentrations. Melanoma cells were harvested from the flasks using 0.25% (*w*/*v*) Trypsin (15090046, Gibco). Human melanoma cells and zebrafish melanoma cells were then seeded into 96-well plates at an initial density of 1 × 10^4^ and 4 × 10^4^ cells per well, respectively. Following a 24-h adhesion period, cells were cultured with medium containing different CLnA isomers, namely JA, PunA, α-ESA, and β-eleostearic acid (β-ESA, C18:3 t9, t11, t13), at different concentrations (5, 10, 20, 40 and 80 µM) for 72 h. Control treatments included either oleic acid (OLA), a monounsaturated fatty acid, or ALA, the non-conjugated counterpart of CLnAs, at different concentrations (5, 10, 20, 40 and 80 µM), as well as a negative control without any additional fatty acid. The treatment concentration and time were determined according to preliminary experiments and previous research [16]. After 72 h of fatty acid treatment, the culture medium was removed, and 100 µL of PrestoBlue (12083745, Fisher Scientific, Pittsburgh, PA, USA) solution, diluted in PBS at a ratio of 1:9 (*v*/*v*), was added to each well. After 1 h of incubation at the cell culture temperature, cell viability was assessed using a Fluoroskan Ascent FL fluorometer (Thermo Scientific, Waltham, MA, USA) at 530/584 nm (excitation/emission) according to the manufacturer’s instructions.

***Assessment of cell death mechanisms underlying CLnA toxicity in melanoma cells.*** The three cell lines were seeded in 96-well plates. Human melanoma cells (A375 and WM266.4) were exposed to 5 µM PunA or JA, while ZMEL1 cells were treated with 20 µM PunA or JA, each in combination with increasing doses of ferroptosis inhibitors, either ferrostatin-1 (fer-1, SML0583, Sigma), α-tocopherol (α-T, 258024, Sigma), or deferoxamine mesylate (DFOM, D9533, Sigma), as well as with the necroptosis inhibitor necrostatin-1(nec-1, 480065, Sigma) and the apoptosis inhibitor ZVAD-FMK (ZVAD, S7023, Selleck Chemicals, Houston, TX, USA). Absolute ethanol was used as a vehicle for α-T, while DMSO was used as a vehicle for fer-1, DFOM, nec-1 and ZVAD. Additional 96-well plates were subjected to either 2.5 µM PunA (A375 and WM266.4 cells) or 10 µM PunA (ZMEL1 cells), in combination with an increasing dose of the ACSL4 inhibitor PRGL493 (HY-139180, MedChemExpress, Monmouth Junction, NJ, USA). Similarly, 0.625 µM PunA was used for the human melanoma cells and 5 µM PunA for ZMEL1 cells, to study the effects of the combination with increasing doses of the GPX4 inhibitors RSL3 (S8155, Selleck Chemicals, Houston, TX, USA) or ML210 (S0788, Selleck Chemicals). DMSO was used as a vehicle for PRGL493, RSL3 and ML210.

### 2.3. Kinetics of ZMEL1 Cell Viability Loss

To determine the optimal treatment concentration and time point to harvest ZMEL1 cells for fatty acid uptake studies and mRNA expression analyses, we assessed cell viability over time under different treatment conditions. ZMEL1 cells were seeded at an initial density of 4 × 10^4^ cells per well into 96-well plates and were exposed to three concentrations (i.e., 10, 20 or 40 µM) of PunA or JA for either 0, 12, 24, 48 or 72 h followed by a viability test.

### 2.4. Fatty Acid Uptake and Metabolic Processing in ZMEL1 Cells

Owing to their lower sensitivity to CLnAs in comparison to the human melanoma cells under investigation, ZMEL1 cells were used to evaluate the uptake and distribution of PunA, JA, or ALA in different fractions, including neutral lipids (NLs), free fatty acids (FFAs), and phospholipids (PLs). Cells were either untreated (control) or treated with 20 μM of PunA, JA or ALA, and were harvested at 0, 4 and 12 h after treatment. Total lipids were extracted using the Bligh and Dyer method [16,38]. The internal standard, consisting of triheptadecanoin (for NLs), nonadecanoic acid (for FFAs), and 1,2-dibehenoyl-sn-glycero-3-phosphocholine (for PLs), was added to each sample to evaluate extraction efficiency. Samples were dried under a nitrogen stream at 30 °C and resuspended in chloroform. Resuspended samples were loaded onto solid phase extraction columns (12102089, Agilent Technologies, Santa Clara, CA, USA) and NL, FFA and PL fractions were eluted with chloroform:2-propanol (2:1, *v*/*v*), diethyl ether:acetic acid (98:2, *v*/*v*) and methanol, respectively [39,40]. The eluted fractions were then evaporated under a nitrogen stream at 30 °C and methylated under alkaline conditions with 0.5 mL of 0.1 M KOH in methanol at 70 °C for 1 h, followed by acidic conditions at 70 °C for 15 min after the addition of 0.2 mL of 1.2 M HCl in methanol. Fatty acid methyl esters (FAMEs) were extracted using 1 mL of hexane. An injection standard, methyl-undecanoate (20-1100-13, Larodan), was added to each sample for accurate measurement during analysis. FAMEs were then injected and separated using a gas chromatograph (Trace 1310, Thermo Fisher Scientific, Waltham, MA, USA) equipped with an autosampler (TriPlusAS, Selangor, Malaysia) and a RT-2560 capillary column (biscyanopropylpolysiloxane, 100 m length, 0.25 mm internal diameter, 0.2 mm film thickness, Restek, Bellefonte, PA, USA). The flow of hydrogen was used as the carrier gas at a constant pressure of 200 kPa. The temperature program for the GC was as follows: the temperature was initially set at 80 °C; it was ramped up to 175 °C at a rate of 25 °C/min and was maintained for 25 min; it was then increased to 200 °C at 10 °C/min and was held for 20 min; it was then increased to 220 °C at 10 °C/min and was held for 5 min; it was then elevated to 235 °C at 10 °C/min with a final hold of 15 min, before being brought back to 80 °C at a rate of 20 °C/min. FAMEs were detected using a flame ionization detector (FID) set at a constant temperature of 255 °C with an air flow of 350 mL/min, hydrogen flow of 35 mL/min, and nitrogen flow of 40 mL/min. A calibration standard consisting of a mixture of 43 pure methyl ester standards (Larodan, Solna, Sweden and Nu-Check Prep, Elysian, MN, USA) was utilized to identify unknown peaks based on their retention times and to quantify them using known concentrations. A PunA methyl ester standard (20-1875, Larodan) of known concentration was used to identify and quantify the PunA peak in each sample. JA was quantified on the basis of the PunA standard; the values were thus expressed in PunA equivalents. Chromatograms were processed using ChromQuest 5.0 software (Thermo Fisher Scientific).

### 2.5. Impact of CLnAs on acsl4 and gpx4 Expression in ZMEL1 Cells

For the assessment of *acsl4* and *gpx4* mRNA expression in ZMEL1 cells, cells were seeded into 6-well plates at an initial density of 1.13 × 10^6^ per well. Once adhered to the plate, cells were treated with 20 μM of JA, PunA, α-ESA, and β-ESA, as well as with OLA and ALA or with DMEM culture medium (as a negative control) for 24 h. After the treatment, total RNA was extracted using the High Pure RNA Isolation Kit (11828665001, Roche, Basel, Switzerland). One microgram of RNA was reverse transcribed into cDNA using the iScript™ cDNA Synthesis Kit (1708891, Biorad, Hercules, CA, USA) according to the manufacturer’s instructions, using an Applied Biosystems SimpliAmp Thermal Cycler (A24811, ThermoFisher). The synthesized cDNAs were amplified by real-time qPCR using the GoTaq qPCR mixture (Promega, Madison, WI, USA) on an Applied Biosystems StepOnePlus Real-Time PCR System (4376600, ThermoFisher) over 40 cycles. Primers used for the qPCR are listed in Supplementary Table S1. The expression levels of target genes were normalized to the expression of housekeeping genes (HKGs), namely β-actin 2 (ACTB2), β-2-microglobulin (B2M), Hypoxanthine-guanine phosphoribosyl transferase (HPRT1) and TATA-binding protein (TBP).

### 2.6. Statistical Analysis

Data are expressed as mean ± standard error of the mean (SEM) of three independent replicates. Statistical analyses were performed with GraphPad Prism 10 software using one-way or two-way ANOVA with Dunnett’s test or Tukey multiple comparison test, when appropriate. Statistical significance relative to the control or another treatment was determined as follows: * *p* ≤ 0.05; ** *p* ≤ 0.01; *** *p* ≤ 0.001; **** *p* ≤ 0.0001.

## 3. Results

### 3.1. CLnAs Are Cytotoxic to Melanoma Cells

**Figure 1:**
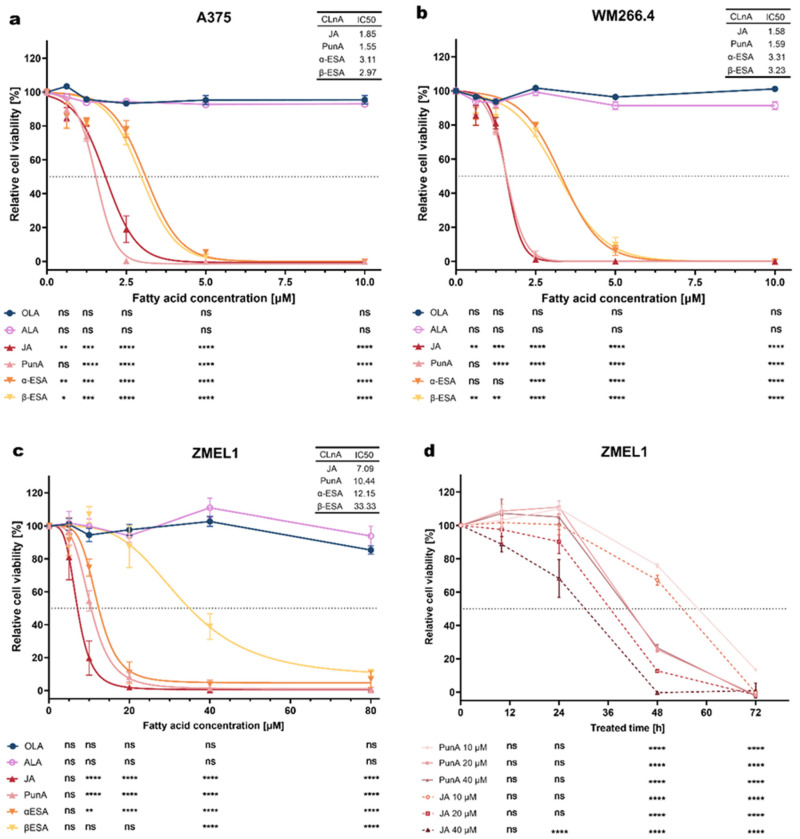
Effects of different fatty acids on the viability of melanoma cell lines of human (A375, WM266.4) or zebrafish (ZMEL1) origin. The A375 (**a**), WM266.4 (**b**) and ZMEL1 (**c**) cell lines were treated for 72 h with varying concentrations of the following fatty acids: oleic acid (OLA), α-linolenic acid (ALA), jacaric acid (JA), punicic acid (PunA), α-eleostearic acid (α-ESA) and β-eleostearic acid (β-ESA). In addition, a kinetic study (**d**) was performed on ZMEL1 cells treated with PunA or JA at 10, 20, or 40 μM over 72 h. Relative cell viability was normalized to control cells that were cultured with medium without added fatty acid, defined as 100%. The horizontal black dotted lines in Figure 1a–d represent the 50% relative cell viability level, indicating the half-maximal inhibitory concentration (IC50). Data are presented as mean ± standard error of the mean (SEM) of 3 independent experiments. Statistical significance was assessed using two-way ANOVA with Dunnett’s test, comparing different concentrations (5, 10, 20, 40, 80 μM) of fatty acid treatments against 0 μM in Figure 1a–c and every time point against 0 h in Figure 1d. ns, not significant, p < 0.05 (*), p < 0.01 (**), p < 0.001 (***), p < 0.0001 (****).

To assess the cytotoxic potential of different fatty acids on melanoma cells, the cell viability across three melanoma cell lines, i.e., A375, WM266.4 and ZMEL1, treated with different concentrations of fatty acids was measured (Figure 1). The human melanoma cell lines (A375 and WM266.4) showed a significant decrease in their viability, even at concentrations as low as 0.625 μM for JA and β-ESA (Figure 1a,b). Complete loss of viability was observed at concentrations of 2.5 μM for PunA in both cell lines, while JA induced complete loss at 2.5 μM in WM266.4 cells and 5 μM in A375 cells. For α-ESA and β-ESA, near-complete loss of viability occurred at 5 μM in both WM266.4 and A375 cells (Figure 1a,b). In A375 cells, the half maximal inhibitory concentrations (IC50) values for JA, PunA, α-ESA, and β-ESA were 1.85, 1.55, 3.11, and 2.97 μM, respectively (Figure 1a). Similarly, the IC50 values for WM266.4 cells were 1.58, 1.59, 3.31, and 3.23 μM for JA, PunA, α-ESA, and β-ESA, respectively (Figure 1b). ALA, a non-conjugated counterpart of CLnAs, as well as OLA, were not cytotoxic to both human melanoma cell lines, even at concentrations up to 10 μM.

For ZMEL1 cells, the four tested CLnAs (JA, PunA, α-ESA, and β-ESA) showed varying levels of toxicity. While β-ESA required a high concentration (40 μM) to induce significant cytotoxicity, the other three CLnAs exhibited significant cytotoxic effects at 10 μM (Figure 1c). The IC50 values for JA, PunA, α-ESA, and β-ESA were determined to be 7.09, 10.44, 12.15, and 33.33 μM, respectively (Figure 1c). Complete loss of cell viability was observed following treatment with 20 µM JA or 40 µM PunA or α-ESA, indicating that JA exhibited the highest cytotoxicity among the four tested CLnA isomers. Conversely, ALA and OLA did not induce significant cytotoxicity on ZMEL1 cells even at a concentration as high as 80 μM (Figure 1c).

Taken together, our results demonstrate that CLnAs are highly cytotoxic for the three melanoma cell lines tested, with JA and PunA being the most potent across both human and zebrafish melanoma cell lines. These results also highlight that ZMEL1 cells are less sensitive to CLnAs, as compared to the tested human melanoma cells.

This reduced susceptibility of ZMEL1 cells enabled the collection of viable cells (see Figure 1c), following exposure to CLnA at concentrations allowing analysing the fate of intracellular CLnAs and their potential metabolites. For this purpose, we performed time course studies evaluating the viability of ZMEL1 cells following PunA or JA treatment to determine optimal treatment concentration and time point for subsequent fatty acid uptake and mRNA expression analyses (Figure 1d). When treated with 10, 20 and 40 μM of PunA or 10 and 20 μM of JA, ZMEL1 cell viability did not exhibit any noticeable decrease within the first 24 h. In contrast, ZMEL1 cells treated with 40 μM JA already showed a significant decrease in viability after 24 h of incubation. After 48 h of treatment, a decline in viability was observed for all tested PunA and JA concentrations. These findings suggest that treating ZMEL1 cells with 20 μM of either PunA or JA for 24 h allows for harvesting cells at an early stage of CLnA-induced stress, before any substantial loss of viability occurs.

### 3.2. Ferroptosis Inhibitors Mitigate CLnA-Induced Toxicity in Melanoma Cells

We hypothesized that CLnAs induce ferroptosis in melanoma cells, similarly to what has been observed in other types of cancer cell lines [15,17,23]. Thus, we exposed melanoma cells to CLnAs in the presence of inhibitors targeting ferroptosis, necroptosis and apoptosis to investigate whether CLnA toxicity was a consequence of one of these three cell death modalities. The three investigated melanoma cell lines were treated with a lethal dose of PunA or JA (5 μM for human melanoma cells and 20 μM for ZMEL1 cells) in the presence of ferroptosis inhibitors.

**Figure 2:**
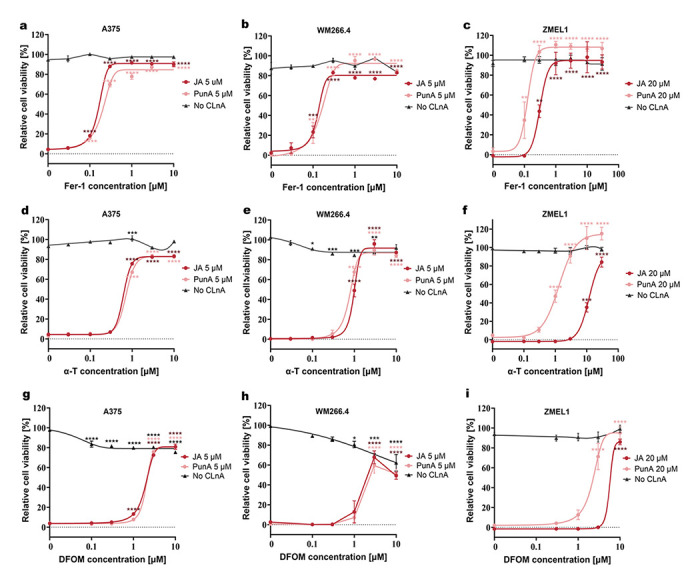
Effects of ferroptosis inhibitors on the CLnA-treated melanoma cells. Viability of A375, WM266.4 and ZMEL1 cells treated with punicic acid (PunA) or jacaric acid (JA) was assessed in the presence of increasing doses of ferrostatin-1 (fer-1; (**a**–**c**)), α-tocopherol (α-T; (**d**–**f**), or deferoxamine mesylate (DFOM; (**g**–**i**)). Relative cell viability was normalized to control cells that were cultured with medium without any added inhibitor or CLnA, defined as 100%. For the cells cultured without CLnA, the culture medium still included the vehicles used for fer-1, α-T, and DFOM. Data are presented as mean ± standard error of the mean (SEM) of 3 independent experiments. Dose-response curves have been fitted to the data. Statistical significance was assessed using two-way ANOVA with Dunnett’s test, comparing different inhibitor concentrations against 0 μM. p < 0.05 (*), p < 0.01 (**), p < 0.001(***), p < 0.0001 (****). Only significant differences are shown.

A dose-dependent prevention of cell death was shown with each of the ferroptosis inhibitors tested (Figure 2). Regarding fer-1, the viability of A375 and WM266.4 cells treated with 5 μM PunA or JA was significantly increased at a dose as low as 0.1 μM (Figure 2a,b). A similar trend was observed in ZMEL1 cells treated with 20 μM PunA or JA, where fer-1 treatment also significantly prevented cell death from doses as low as 0.1 μM and 0.3 μM, respectively (Figure 2c).

Another ferroptosis inhibitor, α-T, also efficiently mitigated CLnA-induced cytotoxicity. The presence of α-T significantly restored the viability of both PunA- and JA-treated human melanoma cells from a concentration of 1 μM (Figure 2d,e). Regarding PunA-treated ZMEL1 cells, the viability was significantly improved at α-T concentrations starting from 1 μM, while JA-treated cells required an α-T dose of 10 μM (Figure 2f).

DFOM also protected the melanoma cell lines from PunA- and JA-induced toxicity. DFOM at 3 µM or 1 µM provided significant protection to A375 cells exposed to 5 µM of PunA or JA, respectively (Figure 2g). In WM266.4 cells, this was observed at 3 µM DFOM in both PunA- and JA-treated (Figure 2h). In ZMEL1 cells treated with PunA or JA, viability was significantly preserved at concentrations of 3 µM and 10 µM, respectively (Figure 2i). Of note, fer-1 and α-T did not display a marked cytotoxicity towards melanoma cells when applied alone (Figure 2a–f). In contrast, a significant loss of viability could be observed in A375 cells and WM266.4 cells from the low doses of 0.1 μM and 1 μM DFOM, respectively (Figure 2g,h). This phenomenon is likely to result from the detrimental impact of iron chelation on cellular iron homeostasis, independently of cell death inhibition, consequently diminishing cell growth, as previously reported [41].

ZMEL1 cells treated with 20 µM JA required higher concentrations of all ferroptosis inhibitors than cells treated with 20 μM PunA to achieve a survival rate above 50%. This phenomenon was not observed in the human melanoma cell lines, emphasizing the differential toxicity of CLnA isomers across melanoma cell lines from different species.

In order to evaluate the potential involvement of necroptosis and apoptosis in CLnA-induced cell death, we tested the impact of the necroptosis inhibitor nec-1 and the apoptosis inhibitor ZVAD. In all three cell lines, nec-1 failed to significantly maintain cell viability even at 10 μM in human melanoma cells and 30 μM in ZMEL1 cells, indicating that necroptosis is not the cell death pathway through which CLnAs exert their cytotoxicity (Figure S2a–c). The apoptosis inhibitor ZVAD significantly mitigated the impact of the apoptosis inducer staurosporine in all three cell lines, starting at a concentration of 30 μM in human melanoma cells and 10 μM in ZMEL1 cells. In contrast, its capacity to restore viability in JA- or PunA-treated cells remained limited (Figure S2d–f), indicating that apoptosis is not the primary mechanism of cell death induced by CLnAs. Taken together, our results indicate that ferroptosis is the main cause responsible for CLnA-triggered cytotoxicity in both human and zebrafish melanoma cells.

### 3.3. GPX4 or ACSL4 Inhibition Influences PunA Cytotoxicity in Melanoma Cells

**Figure 3:**
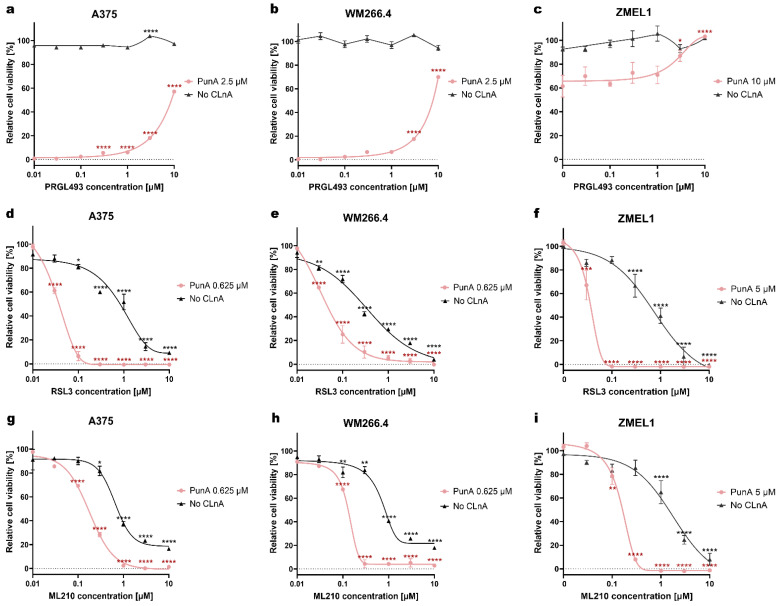
Effects of ACSL4 or GPX4 inhibitors on the PunA-induced cytotoxicity in melanoma cells. Viability of A375, WM266.4 and ZMEL1 cells treated with punicic acid (PunA) was assessed in the presence of increasing doses of PRGL493 (**a**–**c**), RSL3 (**d**–**f**) or ML210 (**g**–**i**). Relative cell viability was normalized to control cells that were cultured with medium without any added inhibitor or CLnA, defined as 100%. For the cells cultured without CLnA, the culture medium still included the vehicles used for PRGL493, RSL3 and ML210. Data are presented as mean ± standard error of the mean (SEM) of 3 independent experiments. Dose-response curves have been fitted to the data. Statistical significance was assessed by two-way ANOVA with Dunnett’s test, comparing different tested inhibitor concentrations (0.03, 0.1, 0.3, 1, 3, 10 μM) against 0 μM. p < 0.05 (*), p < 0.01 (**), p < 0.001 (***), p < 0.0001 (****). Only significant differences are shown.

We next investigated how modulation of ACSL4, which promotes lipid peroxidation and GPX4, which reduces lipid hydroperoxides, influences the cytotoxicity of PunA in the three investigated melanoma cell lines. The viability of the three melanoma cell lines remained largely unaffected when treated with the ACSL4 inhibitor, PRGL493, alone (Figure 3a). However, when combined with 2.5 μM PunA in human melanoma cells or 10 μM PunA in ZMEL1 cells, PRGL493 progressively mitigated the toxicity of PunA as its concentration increased. The cell viability of A375 and WM266.4 cells was significantly higher in the presence of PRGL493 from 0.3 μM and 3 μM, respectively (Figure 3a,b). For the ZMEL1 cells, a significant restraining effect of PRGL493 was observed from a concentration of 3 μM (Figure 3c).

The inhibition of GPX4 drastically enhanced PunA cytotoxicity at sub-lethal doses (0.625 μM for human melanoma cells and 5 μM for ZMEL1) in all three tested melanoma cell lines. RSL3 and ML210 are inhibitors of GPX4, leading to the accumulation of toxic lipid peroxidation products and, subsequently, ferroptotic cell death [42]. In both A375 and WM244.6 cells, RSL3 significantly enhanced the cytotoxic effects of PunA, even at a concentration of 0.03 μM, leading to a marked reduction in cell viability in the presence of 0.625 μM PunA. At a dose of 0.1 μM, RSL3 led to nearly complete cell death in PunA-treated A375 cells, while cells treated with RSL3 alone maintained a viability of 81% (Figure 3d). The same trend was observed in WM266.4 cells, since the cell viability of PunA-treated cells decreased to 26% in the presence of 0.1 μM RSL3, whereas the cells with RSL3 alone kept a viability of 72% (Figure 3e). ML210 showed a similar effect to RSL3 in human melanoma cells. In the case of A375 and WM266.4 cells treated with 0.625 μM PunA, the viability already significantly declined with 0.1 μM ML210 (Figure 3g,h). With 1 μM ML210, PunA-treated A375 cells showed almost complete loss of viability, while cells without PunA treatment experienced a significant reduction in viability of 63% (Figure 3g). As for the WM266.4 cell line, PunA-treated cells showed near-complete loss of viability with 0.3 μM ML210, while the viability of cells without PunA remained around 84% (Figure 3h).

In PunA-treated ZMEL1 cells, RSL3 led to a complete loss of viability at the low dose of 0.1 μM, while cells without PunA maintained 88% viability under the same dose of RSL3. A dose of 10 μM RSL3 was needed to reach a complete loss of viability of ZMEL1 cells in the absence of PunA (Figure 3f). Regarding ML210, the low dose of 0.3 μM led to a dramatic drop in viability down to only 7.9% when combined with 5 μM PunA, whereas cells treated with the same dose of ML210 alone retained 85% viability (Figure 3i). These results indicate that GPX4 inhibition significantly enhances PunA toxicity across all tested melanoma cell lines.

### 3.4. CLnAs Show Efficient Cellular Uptake and Differential Distribution in Different Fractions

**Figure 4:**
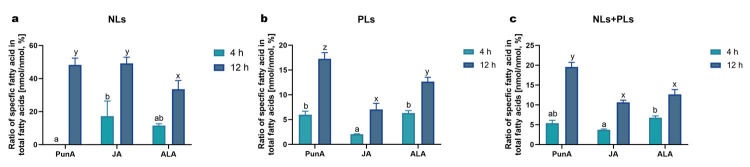
Cellular uptake and distribution of PunA, JA or ALA in ZMEL1 cells. Enrichment in punicic acid (PunA), jacaric acid (JA), and α-linolenic acid (ALA) in ZMEL1 cells treated with 20 μM of each fatty acid for 4, or 12 h is shown in neutral lipids (NLs; (**a**)), phospholipids (PLs; (**b**)), and both fractions combined (NLs + PLs; (**c**)). All values at 0 h were below the limit of detection. *Data are presented as mean ± standard error of the mean (SEM) of 3 independent cultures and are expressed as the ratio of PunA, JA or ALA amount to the total amount of all fatty acids in each fraction (%, nmol/nmol). Statistical significance was assessed using two-way ANOVA with Tukey’s multiple comparisons. Statistical differences among PunA, JA, and ALA, at both 4 h and 12 h, are presented by the letters a, b and x, y, z above the bars, respectively.*

To further elucidate how CLnAs trigger ferroptosis in melanoma cells, the cellular uptake of PunA and JA and their distributions among NL, FFA, and PL fractions were investigated. A condition with ALA was added as a control. Due to limitations in the sensitivity of the fatty acid quantification method, this experiment was restricted to the ZMEL1 cell line, which maintains viability for at least 12 h in the presence of high doses of CLnAs (i.e., 20 μM PunA or JA), ensuring sufficient intracellular fatty acids for accurate quantification. The cells were treated for either 4 or 12 h. The added fatty acids were recovered in NLs and PLs, while their presence in the FFA fraction was negligible and therefore not considered. Enrichment of a given fatty acid was calculated as its amount normalized to the total amount of fatty acids in each fraction (in nanomoles) (Figure 4). The gradual enrichment in PunA, JA and ALA in the NL and PL fractions indicated their efficient uptake and progressive accumulation in ZMEL1 cells over time (Figure 4). Globally, after 12 h of incubation, each of the tested fatty acids represented between 10% and 20% (nmol/nmol) of all cellular fatty acids (Figure 4c), with higher percentages in NL fractions (30% to 50%) than in PL fractions (7% to 18%) (Figure 4a,b). Regarding the PL fraction, which is critical in the ferroptosis process [29], the enrichment was greater for PunA than for JA at both 4 h and 12 h of incubation, indicating that the higher toxicity of JA in ZMEL1 cells is not due to a greater accumulation in cell membranes.

### 3.5. CLnA Isomers Differentially Modulate ACSL4 and GPX4 Expression in ZMEL1 Cells

**Figure 5:**
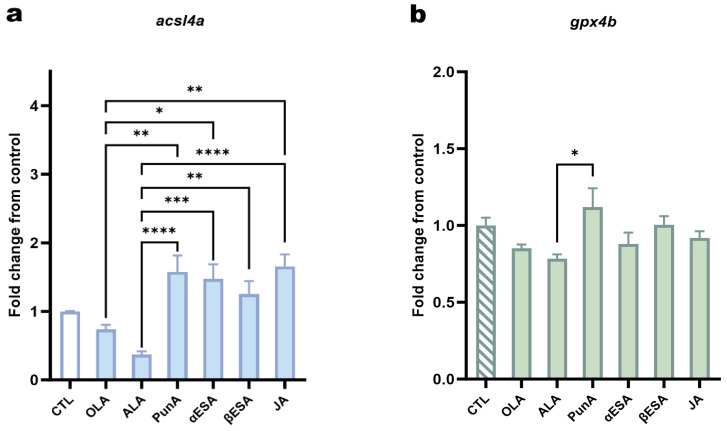
Modulation of *acsl4a* and *gpx4b* gene expression in ZMEL1 cells treated with fatty acids. The fold changes in relative quantity (RQ) were calculated for *acsl4*a (**a**) or *gpx4*b (**b**) in cells exposed to 20 μM of a tested fatty acid for 24 h, relative to the control cells, which were cultured with DMEM without any added fatty acid. The following fatty acids were tested: α-linolenic acid (ALA), oleic acid (OLA), punicic acid (PunA), α-eleostearic acid (α-ESA), β-eleostearic acid (β-ESA) and jacaric acid (JA). The RQ was calculated as RQ = 2^−ΔCt^, the ΔC_t_ value representing the difference in C_t_ value between the target gene and a panel of reference genes, which are actin beta 2, beta-2-microglobulin, hypoxanthine phosphoribosyl transferase 1 and TATA-binding protein. Data are presented as mean ± standard error of the mean (SEM) of 3 independent experiments (N = 3, n = 3). Significance was assessed by one-way ANOVA with Tukey’s multiple comparison test among treatments. Significant differences between the fatty acid-treated groups and the control are marked above the columns. p < 0.05 (*); p < 0.01 (**); p < 0.001 (***), p < 0.0001 (****). Only significant differences are shown.

Given the critical roles of ACSL4 and GPX4 in ferroptosis, we investigated how CLnA treatment affects their expression. The experiments were conducted on ZMEL1 cells, which tolerate higher CLnA doses. Both ACSL4 and GPX4 have two paralogues in zebrafish, namely ACSL4a/ACSL4b and GPX4a/GPX4b, respectively [43,44]. The RNA-seq data available on the GEO database (Series No.: GSE151679) suggest that *acsl4a* and *gpx4b* are the dominant isoforms functioning in ZMEL1 cells [36]. Accordingly, *acsl4a* and *gpx4b* exhibited substantially higher mRNA relative quantity (RQ) levels compared to *acsl4b* and *gpx4a* in control ZMEL1 cells without added fatty acids (Figure S3). Given that RQ values of *acsl4b* and *gpx4a* were relatively low, the subsequent studies primarily focused on the expression of *acsl4a* and *gpx4b* in ZMEL1 cells following fatty acid treatment. ZMEL1 cells treated with PunA, α-ESA and JA showed significant differences in *acsl4a* expression, as compared to both the ALA- and OLA-treated cells (Figure 5a). The β-ESA treatment induced the least upregulation of *acsl4a* expression, which was significant when compared to ALA-treated cells (Figure 5a). As for the expression of *gpx4b*, none of the CLnAs could induce a change compared to control cells (Figure 5b). A slight but significant upregulation was, however, observed in cells treated with PunA compared to ALA-treated cells (Figure 5b).

## 4. Discussion

Given their higher susceptibility to oxidation, as compared to their non-conjugated counterparts, CLnAs have shown promising anti-cancer effects in several cancer types. However, their potential activity against melanoma remains unexplored. Here we show for the first time that CLnAs exert significant cytotoxic effects on melanoma cells of both human (A375 and WM266.4) and zebrafish (ZMEL1) origin. Still, the tested human melanoma cell lines displayed a markedly higher sensitivity to CLnAs, as reflected by their IC50 values, which were approximately 4- to 10-fold lower than those observed for the zebrafish melanoma cell line under investigation. The reduced sensitivity of ZMEL1 cells to CLnAs might be driven by their stronger antioxidant defence combined with the temperature-dependent kinetics of lipid peroxidation. Zebrafish melanoma cells, cultured at their optimal temperature of 28.5 °C, naturally enrich their membranes with PUFAs to maintain fluidity [45]. To counteract the inherent risk of spontaneous lipid peroxidation in their high-PUFA membranes, these cells utilize robust basal antioxidant defences, such as upregulated superoxide dismutase (SOD) [46]. Furthermore, since lipid peroxidation is a temperature-dependent radical chain reaction, the propagation of lethal lipid hydroperoxides is thermodynamically slower at 28.5 °C than at the 37 °C condition used for human cells. This slower kinetic rate maintains a stable baseline “peroxide tone” [47,48]. Consequently, higher concentrations of exogenous CLnAs are required to overwhelm these biological and thermodynamic constraints to trigger ferroptosis in ZMEL1 cells.

To assess if CLnAs induce melanoma cell death through ferroptosis, as they do in other cancer cell models [11], we utilized three distinct inhibitors targeting key mechanistic nodes of lipid peroxidation. Iron, as both a lipoxygenase cofactor and a Fenton reaction catalyst, was chelated using DFOM [21,49]. Peroxyl radical propagation was inhibited or prevented with the lipophilic antioxidant α-T and the alkoxyl radical scavenger fer-1 [50,51]. In contrast to inhibitors of necroptosis and apoptosis, all three ferroptosis inhibitors effectively restored melanoma cell viability, unequivocally identifying ferroptosis as the key cell death program triggered by CLnAs in melanoma cells.

The toxicity of CLnAs towards melanoma cells showed clear isomer-specific patterns. In human melanoma cell lines, PunA and JA were the most potent compounds, whereas α-ESA and β-ESA showed weaker but comparable toxic effects. In ZMEL1 cells, JA remained the most cytotoxic isomer, followed by PunA and α-ESA, while β-ESA exhibited only limited cytotoxicity. The greater cytotoxicity of JA and PunA may be linked to their c,t,c geometric configuration (8, 10, 12 for JA; 9, 11, 13 for PunA), which enhances their oxidative susceptibility [52] and thereby their ability to induce ferroptosis. Nevertheless, the relationship between CLnA configuration and cytotoxicity appears to strongly depend on cell context. In breast cancer cells, JA was reported to potently induce ferroptosis (IC50 1.8 μM), while PunA exhibited weaker activity (IC50 19.4 μM) [23]. In DLD-1 human colorectal adenocarcinoma cells, JA was also found to be the most potent isomer after a 24-h treatment, followed by α-ESA, PunA, and β-ESA [53]. These findings indicate that the cytotoxicity differences among CLnA isomers are not restricted to melanoma cells. The disparity in sensitivity to CLnAs across studies may be linked to reported variations in the activity of enzymes involved in fatty acid incorporation into polar lipids, fatty acid oxidation, and ferroptosis across different types of cancer cells [54,55]. As an example, Beatty et al. demonstrated that CLnA-induced ferroptosis in breast cancer cells mostly relies on ACSL1 [23], whereas our results point to ACSL4 as a key contributor to ferroptosis induction in melanoma cells. Taken together, these findings highlight (i) the importance of isomer geometric configuration, which governs their intrinsic physicochemical properties, such as oxidative stability, and (ii) the influence of cell-specific enzymatic machineries, such as ACSL isoforms, in shaping cancer cell resistance to CLnAs.

The differential cytotoxicity of CLnA isomers was further substantiated by their distinct responses to ferroptosis inhibitors. In A375 and WM266.4 cells, comparable concentrations of ferroptosis inhibitors (i.e., DFOM, α-T, or fer-1) were required to restore viability in both JA- and PunA-treated cells, aligning with the similar toxicity levels of these two isomers in human melanoma cells. In contrast, ZMEL1 cells required higher inhibitor concentrations to mitigate JA-induced cell death than PunA-induced cell death, corroborating the higher cytotoxicity of JA in this zebrafish cell line. All these findings underscore the variable cytotoxicity of CLnA isomers towards melanoma cells, with JA and PunA emerging as the most potent inducers of ferroptosis in melanoma cells among the four tested CLnA isomers. This positions JA and PunA as promising candidates for future in vivo studies.

Ferroptosis is triggered by the accumulation of PUFA-derived lipid peroxides. ACSL4 indirectly facilitates their formation through the incorporation of these fatty acids into the phospholipids, while GPX4 reduces these peroxides and mitigates ferroptotic cell death [56]. Our study demonstrates that CLnA toxicity in melanoma cells is significantly influenced by the functional activity of these two enzymes. Specifically, the inhibition of ACSL4 significantly reduced the toxicity of PunA, whereas the inhibition of GPX4 markedly enhanced it.

The lipid analysis revealed that PunA and JA were effectively incorporated into PLs and NLs in ZMEL1 cells. Even though JA was more cytotoxic than PunA to the ZMEL1 cells, its accumulation in the PL fraction was lower. In contrast, both isomers extensively accumulated in the NL fraction, up to a similar extent after 12 h of incubation. The significant incorporation of JA and PunA into triglycerides, presumably stored within lipid droplets (LDs), presents a complex mechanistic question. On one hand, LDs are often regarded as protective organelles that shield the PUFAs from lipid peroxidation [57,58]. Indeed, studies have shown that exceeding the buffering capacity of triglyceride storage in LDs leads to PUFA-induced ferroptosis [40]. On the other hand, a growing body of evidence suggests that LDs can also act as pro-ferroptotic platforms [57,59]. For example, Beatty et al. demonstrated that α-ESA accumulates in LDs in breast cancer cells where it may act as a potent source of lipid ROS, which can then propagate to cellular membranes [23]. Similarly, Lange et al. recently revealed that while LDs typically sequester PUFAs to prevent damage, the loss of the LD-localized antioxidant FSP1 transforms PUFA-rich LDs into sites of active lipid peroxidation that initiate ferroptosis [59]. Considering melanoma cells, it remains to be determined whether CLnA cytotoxicity is mainly driven by their incorporation into NLs or whether their integration into PLs, where peroxidation can directly trigger ferroptosis, is the dominant mechanism.

The greater cytotoxicity of JA to ZMEL1 cells as compared to PunA might be due to the higher susceptibility of JA to oxidation in the cellular environment [18]. As a consequence, a small amount of JA in the membrane would be sufficient to trigger cell death. Alternatively, JA accumulation in LDs could serve as a localized hub for initiating the ferroptotic cascade, as previously proposed for α-ESA [23]. Finally, a combination of both mechanisms cannot be ruled out based on available data.

The metabolic trafficking of CLnAs into either PLs or NLs is necessarily preceded by their activation to acyl-CoA esters, a process catalyzed by ACSL enzymes. We found that CLnA treatment led to a significant upregulation of *acsl4a* expression when compared to cells treated with ALA or OLA, indicating a specific transcriptional response to these pro-ferroptotic lipids. Interestingly, a significant change was not observed when comparing to untreated control cells. This is not unprecedented, as a similar lack of significant *acsl4* transcriptional change has been reported in other models of PUFA-induced ferroptosis [40]. This suggests that the basal enzymatic activity of ACSL4, rather than its dramatic transcriptional induction, may be the rate-limiting factor for mediating CLnA toxicity. This conclusion is supported by the fact that the inhibition of ACSL4 effectively mitigated the cytotoxic effects of CLnAs in melanoma cells, unequivocally highlighting the critical role of this enzyme in activating these fatty acids to drive ferroptosis.

Regarding *gpx4b*, the dominant *gpx4* isoform in ZMEL1 cells, our results showed that its expression was not significantly altered by CLnA treatment compared to the control or to cells treated with OLA or ALA, apart from a slight upregulation induced by PunA compared to ALA. The minimal transcriptional alteration observed for *gpx4b* aligns with previous reports, showing that GPX4 protein levels remain unaffected in breast cancer cells treated with αESA or JA [15,40]. This collective evidence indicates that CLnAs neither induce ferroptosis by suppressing GPX4 expression nor trigger significant upregulation of this key antioxidant enzyme in response to the resulting lipid peroxidation. These findings actually align with the saturation of the antioxidant capacity of the GPX4 system, which is ultimately overwhelmed by the tide of CLnA-derived lipid hydroperoxides, thereby leading to ferroptosis [23,60].

While GPX4 is a key oxidation defender, it is important to recognize that other antioxidant systems, such as the FSP1/CoQ10 axis, DHCR7 and BH4-GCH1, also contribute to mitigating ferroptosis [17,61]. Thus, although CLnAs do not significantly alter *gpx4* expression in melanoma cells, the possibility remains that they modulate the expression levels of other players within this intricate antioxidant network. In this context, future investigations could explore whether early pro-survival programs, such as the Nrf2 antioxidant program, are transiently triggered by CLnAs, and whether varying abilities to activate this network contribute to the differential cytotoxicity observed among the isomers [62,63].

Zebrafish has emerged as an important model in cancer research, particularly in melanomas [64]. The consistent findings regarding cytotoxicity and the modulation of the ACSL4/GPX4 axis across both human and zebrafish melanoma cells underscore shared mechanisms, highlighting the translational relevance of the zebrafish model. Interestingly, the fluorescently labelled ZMEL1 cell line developed by Heilmann et al. has the potential to track live formation of metastases in the transparent Casper zebrafish [65]. Validating our findings using this zebrafish melanoma model would be a critical next step to observe tumour response to CLnA treatments.

## 5. Conclusions

Our study demonstrates that CLnA isomers (i.e., PunA, α-ESA, β-ESA, and JA) exhibit potent cytotoxicity towards melanoma cells, with JA and PunA being the most effective. The cytotoxicity of CLnAs is driven by ferroptosis, with ACSL4 and GPX4 serving as critical regulators. The combined use of a GPX4 inhibitor with a CLnA represents a promising therapeutic strategy to potentiate the anti-melanoma effects of these compounds. The consistency of these findings across both human and zebrafish cells underscores a shared ferroptotic mechanism and highlights the translational relevance of the zebrafish model for these compounds.

## Data Availability

The original contributions presented in this study are included in the article/Supplementary Materials. Further inquiries can be directed to the corresponding author.

## Supplementary Materials

The following supporting information can be downloaded at: https://www.mdpi.com/article/10.3390/antiox15030360/s1. Figures S1: Chemical structures of oleic acid, α-linolenic acid, and conjugated linolenic acid isomers.; Figures S2: Effects of apoptosis and necroptosis inhibitors on CLnA-treated melanoma cells; Figures S3: mRNA relative quantity (RQ) of acsl4 and gpx4 isoforms in control ZMEL1 cells; Table S1:Forward and reverse primers for target genes and housekeeping genes (HKGs) used for RT-qPCR analyses.

## Abbreviations

The following abbreviations are used in this manuscript:

α-ESA	α-eleostearic acid
ALA	α-linolenic acid
α-T	α-tocopherol
β-ESA	β-eleostearic acid
ACSL4	Acyl-CoA synthetase long-chain family member 4
CLnA	Conjugated linolenic acids
PunA	Punicic acid
DFOM	deferoxamine mesylate
fer-1	ferrostatin-1
GPX4	glutathione peroxidase 4
JA	jacaric acid
LPCAT	lysophosphatidylcholine acyltransferase
nec-1	necrostatin-1
OLA	oleic acid
PL	phospholipid
PUFA	polyunsaturated fatty acid
RQ	relative quantity
