# Supplementary material for: Conjugated Linolenic Acids Induce Ferroptosis in Human and Zebrafish Melanoma Cells
Source: Antioxidants (Basel). 2026 Mar 12;15(3):360. doi: 10.3390/antiox15030360 (PMC13024497; doi:10.3390/antiox15030360)
Supplement: Supplementary file 1 [file antioxidants-15-00360-s001.zip › antioxidants-4187765-supplementary.pdf]

a

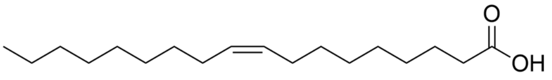

OLA
(C18:1 c9)

b

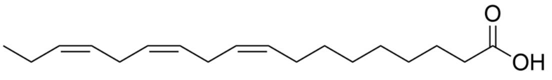

ALA
(C18:3 c9, c12, c15)

c

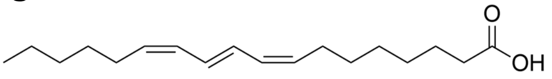

JA
(C18:3 c8, t10, c12)

d

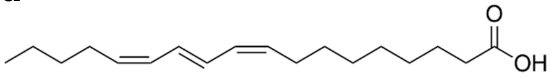

PunA
(C18:3 c9, t11, c13)

e

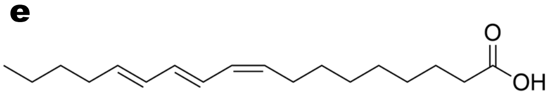

α-ESA
(C18:3 c9, t11, t13)

f

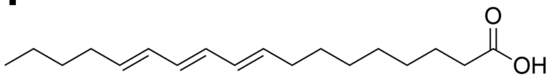

β-ESA
(C18:3 t9, t11, t13)

Supplementary Figure S1. Chemical structures of oleic acid, α-linolenic acid, and conjugated linolenic acid isomers. The fatty acids shown are: oleic acid (OLA, a), α-linolenic acid (ALA, b), jacaric acid (JA, c), punicic acid (PunA, d), α-eleostearic acid (α-ESA, e) and β-eleostearic acid (β-ESA, f).

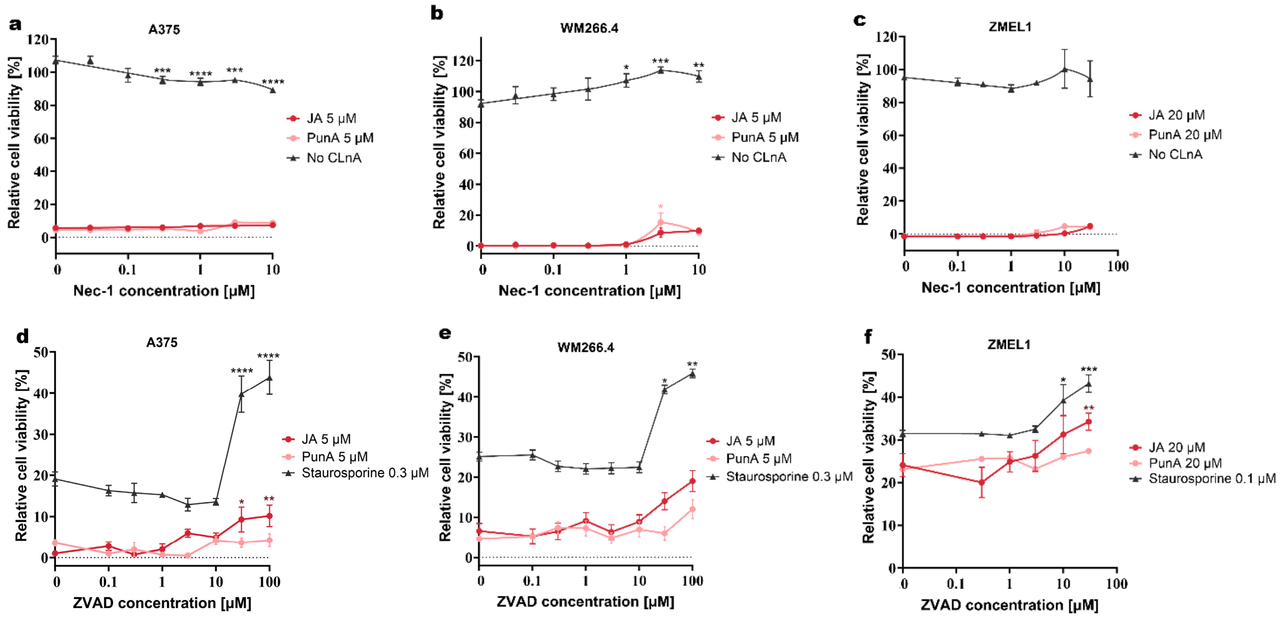

Supplementary Figure S2. Effects of apoptosis and necroptosis inhibitors on CLnA-treated melanoma cells. Viability of A375, WM266.4 and ZMEL1 cells treated with punicic acid (PunA) or jacaric acid (JA) was assessed in the presence of increasing doses of necrostatin-1 (nec-1; a-c) and Z-VAD-FMK (ZVAD; d-f). *Relative cell viability was normalized to control cells that were cultured with medium without any added inhibitor or CLnA, defined as 100%. For the cells cultured without CLnA, the culture medium still included the vehicles used for nec-1 and ZVAD. Data are presented as mean ± standard error of the mean (SEM) of 3 independent experiments. Dose-response curves have been fitted to the data. Statistical significance was assessed using two-way ANOVA with Dunnett's test, comparing different inhibitor concentrations against 0 µM. p<0.05(*), p<0.01(**), p<0.001(***), p<0.0001(****). Only significant differences are shown.*

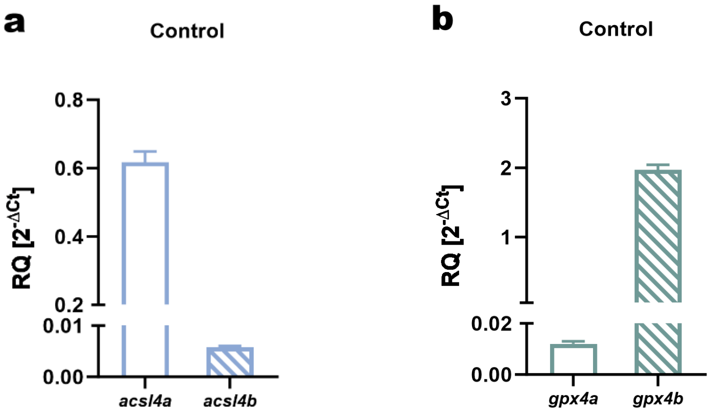

Supplementary Figure S3. mRNA relative quantity (RQ) of *acsl4* and *gpx4* isoforms in control ZMEL1 cells. The RQ values of *acsl4a* and *acsl4b* (a), and *gpx4a* and *gpx4b* (b) were determined in ZMEL1 cells cultured with DMEM without any added fatty acid. *The relative quantity (RQ) value was calculated as RQ =* $2^{-\Delta C_t}$*, the* ΔC_t *value representing the difference in* C_t *value between the target gene and a panel of reference genes, which are actin beta 2, beta-2-microglobulin, hypoxanthine phosphoribosyl transferase 1and TATA-binding protein. Data are presented as mean ± standard error of the mean (SEM) of 3 independent experiments (N=3, n=3).*

Supplementary Table S1. Forward and reverse primers for target genes and housekeeping genes (HKGs) used for RT-qPCR analyses.

	Gene	Accession No.	Gene Forward primer 5'-3'	Reverse primer 5'-3' Source
Target genes	ACSL4a	NM_200649.1	AGCAGGCACTATCACCGAAG	CATTAGGGCCACCGATGAGG
	ACSL4b	NM_001099739.1	GTGTTTCTGCCCCATTGCTG	CCCATGGCAACATTTGGTCC
	GPX4a	NM_001346537	GTGGGATCATTGGTGCAACG	AGCCAGGATGCGTAAACCTC
	GPX4b	NM_001030070	CTGCAACCAGTTCGGAAAGC	CCCAGTGTTCCTCTGCCTTT
HKGs	ACTB2	NM_181601.5	GCAGAAGGAGATCACATCCCTGGC	CATTGCCGTCACCTTCACCGTTC
	B2M	NM_131163.2	ACCTACACATGGGAGCCTGA	GCCGGATCTGCAAGAGTGTT
	HPRT1	NM_212986.2	ATCCGCCTCAAGAGTTACCAAAATG	ATCCAACGCTCCTCGGTGTC
	TBP	NM_200096.1	ACCCACCAGCAGTTTAGCAG	GATTGCGTAGGTCACCCCAG

ACSL4, Acyl-CoA synthetase long-chain family member 4; GPX4, Glutathione peroxidase 4; ACTB2, actin beta 2; B2M, beta-2-microglobulin; HPRT1, Hypoxanthine phosphoribosyl transferase; TBP, TATA-binding protein.
